# SARS-CoV-2 and dengue virus coinfection in an adult with beta-thalassemia (trait): A case report from Bangladesh with literature review

**DOI:** 10.1016/j.heliyon.2021.e08229

**Published:** 2021-10-20

**Authors:** Md Rezaul Hossain, Monira Sarmin, Hafizur Rahman, Lubaba Shahrin, Zannatun Nyma, Tahmeed Ahmed, Mohammod Jobayer Chisti

**Affiliations:** a Nutrition and Clinical Services Division (NCSD), icddr,b; b Clinical Hematology & Cancer Biology, Laboratory Sciences & Services Division (LSSD), icddr,b

**Keywords:** COVID-19, SARS-CoV-2, Dengue, Beta-thalassemia, Hemoglobinopathies, Bangladesh

## Abstract

**Introduction:**

Coinfections are common in pandemics, however not in recorded patients with hemoglobinopathies. The Coronavirus Disease 2019 (COVID-19) pandemic struck Bangladesh at the beginning of March 2020, which is also an apt period for endemic Dengue fever in this monsoon region.

**Case report:**

We report a 30-year-old man with hemoglobinopathies coinfected with Severe Acute Respiratory Syndrome Coronavirus 2 (SARS-CoV-2) and Dengue virus. Dengue virus was detected by Enzyme-linked Immunosorbent Assay (ELISA). COVID-19 was confirmed by Reverse-transcription Polymerase Chain Reaction (RT-PCR) and Hemoglobin Electrophoresis revealed heterozygous beta-thalassemia or thalassemia trait. The patient was treated successfully at Dhaka Hospital in icddr,b during COVID-19 emergency response with symptomatic supportive treatment for COVID-19 and appropriate fluid therapy for dengue fever in response to daily hematocrit level. The patient's repeated RT-PCR for COVID-19 on day-21 became negative. For thalassemia, the patient was advised to have genetic counseling and family screening on discharge.

**Conclusion:**

The possibility of coinfection between COVID-19 and Dengue fever may be considered in a COVID-19 patient with unremitting fever especially in an area where Dengue fever is epidemic that may further help to attain appropriate management of the patient.

## 1 Introduction

Pandemic disease with an infectious agent alone can cause substantial deaths, but when associated with other co-infection mortalities is enormously high. Many deaths occurred during the Spanish Flu, back in 1918 was due to subsequent co-infections. Patients were vulnerable to coinfection between other species along with causative respiratory viral pathogen, which led to increased disease severity and death [1]. After a century since the Spanish Flu, coinfection also remains potentially lethal during the recent 2019 coronavirus disease (COVID-19) pandemic, caused by severe acute respiratory syndrome coronavirus 2 (SARS-CoV-2), which was emerged from Wuhan, China [2, 3]. Zhou and colleagues recently showed that among COVID-19 patients about 50 percent of deaths included secondary bacterial infections [4]. Therefore 71% of admitted COVID-19 patients received antimicrobial treatment, empirically, to prevent secondary or co-infection, despite species’ antimicrobial sensitivities[1]. Some fungal coinfection with SARS-CoV-2 also have been reported by Chen et al. [5] While coinfections are proven to be significantly associated with the severity of respiratory illnesses, they are understudied during major respiratory infection outbreaks, especially coinfection of viruses from two different groups that were unexplored [1]. Furthermore, COVID-19 emerged during the monsoon season, when dengue fever is epidemic in most tropical countries, including Bangladesh [6]. Any of the arbovirus serotypes (among four) that are transmitted by the Ades mosquito cause dengue fever. Dengue fever is the most common and rapidly spreading disease in recent years, and it is widespread in resource-poor parts of the world [6, 7]. For Bangladesh, in particular, there was a large rise in the number of cases of dengue fever that occurred across the country in 2019, posing major public health concerns [6]. Here we report a case, coinfected with two different viruses, simultaneously, from Coronavirus and Flavivirus groups, and managed successfully.

## 2 Case report

*Case Presentation:* A 30-years-old gentleman of middle socio-economic class, living in an apartment with his wife and had no travel history within the last 14 days, presented at the Dhaka Hospital of icddr,b's (formerly known as the International Centre for Diarrhoeal Disease Research, Bangladesh) COVID-19 emergency response provisional tent hospital on 19 June 2020. Since 15 June 2020 (4 days before hospital admission), the patient was found to have COVID-19 confirmed by reverse-transcription polymerase chain reaction (RT-PCR) tested at icddr,b. The patient was receiving medicine after being positive for SARS-CoV-2 according to the COVID-19 treatment protocol practiced in icddr,b. His medication included oral Paracetamol, Ivermectin 12 mg once, Doxycycline, Famotidine, Zinc, Vitamin C, and D. The patient was admitted on 19 June 2020 with complaints of fever for 11 days, the highest recorded temperature was 39.4 °C (102.92°F), associated with no chills and rigors and headache for 2 days. The patient also complained of generalized body-ache for the last 2 days and vomited fresh blood for an episode, at home, on the morning of hospital admission. On admission, the patient's weight was 85 kg (187.39 lbs.) and BMI was 31.2 kg/m^2^. The patient is a non-smoker, non-alcoholic, and had no co-morbidities.

On admission his temperature was 38.6 °C (101.48°F), pulse was 76 beats/minute and blood pressures were 110/79 mm of Hg. Though breathing effort (respiratory rate 24/minute) was mildly increased, the patient was well-maintaining oxygen saturation in room air (SpO_2_ was 99% in room air) (Figure 3). The patient presented with no sign of dehydration. Systematic examinations did not show any abnormal findings.

*Differential Diagnosis:* We set out our differential diagnosis based on the presentation of the case, including COVID-19 with stress ulcer or either Disseminated Intravascular Coagulation (DIC) or Dengue. We conducted relevant laboratory investigations later to reach the final diagnosis, however, the unavailability of upper gastrointestinal (GI) endoscopy remained one of our limitations in excluding any possibility of GI ulcer. Furthermore, we were unable to rule out COVID-19-related frailty, which could have increased the patient's morbidity, as discussed by Van Schaik Sharon and DeWitte, who compared it to the historical context of the Black Death pandemic in the 14th century [8].

[Informed written consent from the patient has been obtained for reviewing the case report and publication. The patient's identity was removed from all data. icddr,b research administration approval was sought and granted for publication in a peer-reviewed journal.]

## 3 Methods

**Figure 1:**
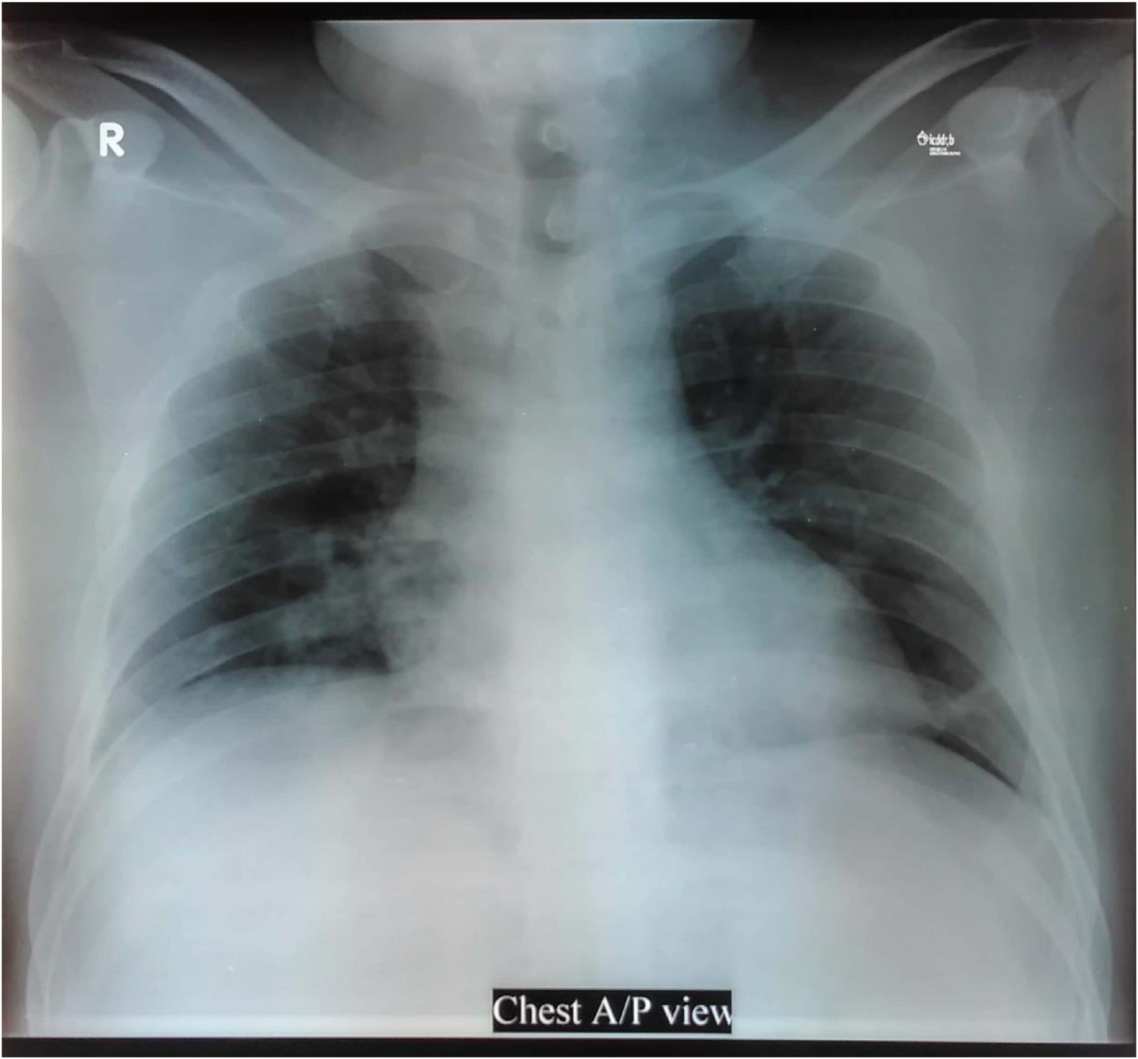
Chest X-ray anteroposterior view showing ill-defined ground-glass opacification (GGO) in the lower left and right lung zone, with an overall score of 2.

*Investigations:* Before admission, the patient was tested positive for COVID-19 by RT-PCR. Upon admission to chest X-ray, COVID-19 radiological pneumonia was suggested by ill-defined ground-glass opacification (GGO) in the lower left and right lung areas (Figure 1). Although a High-resolution computed tomography (HRCT) of the chest was more reliable for the case, we performed a chest X-ray of the patient to evaluate lung field involvement within our limitations. According to the X-ray score system for COVID-19 patients, suggested by Wong et al., the score was 1 (extent of involvement <25%) for each lung with a global score of 2 [9, 10]. Considering the suspicious symptoms of vomiting with fresh blood and several days of high fever, the blood specimen for detecting Dengue virus was sent as the patient was presented during the year when Dengue fever is endemic here in Bangladesh [11].

*Detection of Dengue virus:* The patient's serum was tested by ELISA (using the Panbio™ Dengue Duo IgM Capture and IgG Capture ELISA kit by Abbott, Chicago, Illinois, the United States, which has a serological sensitivity of 94.7% and specificity of 100%) at icddr,b serodiagnostic laboratory according to the manufacturer's instructions for anti-dengue IgM and IgG [28]. There were >40 IgM antibodies in the sample and considered positive for recent dengue virus infection, whereas IgG was not.

**Figure 2:**
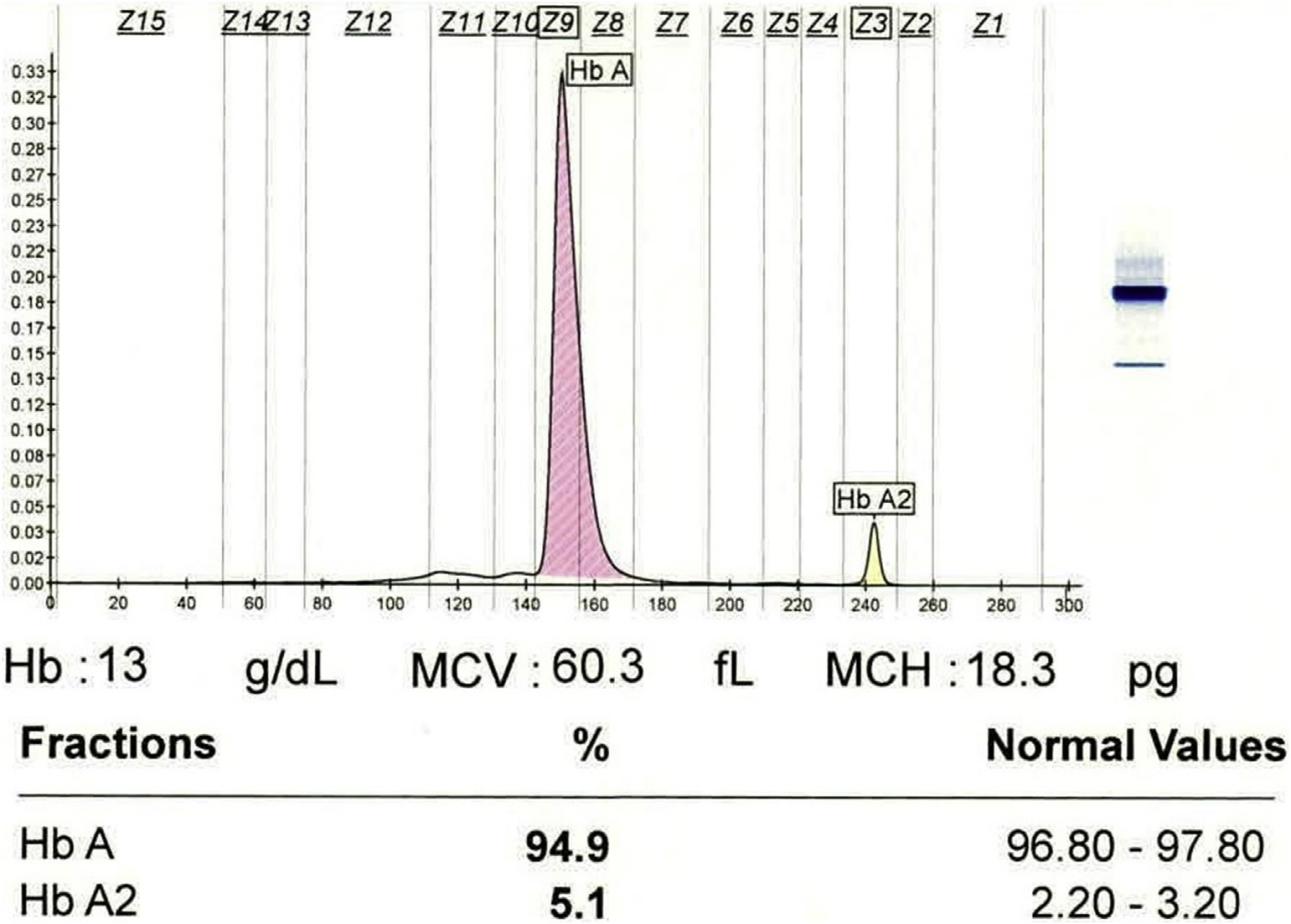
Hemoglobin Electrophoresis showing Hb A2 is 5.1% (HbA2 >3%% is characteristic of heterozygous beta-thalassemia (trait).

*Detection of Hemoglobinopathies:* Complete Blood Count (CBC) on admission showed no thrombocytopenia (platelet was 170,000/μL) or leucopenia (Total leucocyte was 5560/μL). The lower border of hemoglobin (12.8 gm/dL as lowest, after 24 h of admission), mean corpuscular hemoglobin concentration (MCHC) (around 30% in all four reports) and mean corpuscular volume (MCV) around 60 femtolitres (fl) in all four reports were less than the lower margin of reference value 76.0 fl and peripheral blood smear showed hypochromic microcytic anemia. However, being in a recourse poor setting we were unable to assess the patient's serum iron study and serum ferritin to draw an accurate conclusion. To address the issue of lowered red cell indices we performed further hemoglobin electrophoresis of the patient to rule out hemoglobinopathies (Table 1). Hemoglobin Electrophoresis by Capillary method revealed heterozygous Beta-thalassemia or beta-thalassemia trait (HbA2 >3.5 is the diagnostic hallmark of heterozygous beta-thalassemia) (Figure 2). All other blood cell counts ranged within the reference values for the adult (male).

**Table 1:** Laboratory Investigation reports.

Laboratory Data					
Variable	Reference Range, Adults	On Admission	After 24 h	Hospital Day 3	On discharge
Days after symptom onset		11	12	14	15
Blood Group and Rh type		B (+) ve			
Hemoglobin (gm/dL)	13.3–17.0	13.6	12.8	13.1	13.0
Total RBC count (10^12^/L)	4.2–6.2	7.4	7.01	7.13	7.10
Hematocrit/PCV (%)	40.0–52.0	44.9	42.8	43.3	42.9
Total white-cell count (per μL)	4000–11000	5560	3410	4080	4380.0
Differential count (%)					
Neutrophils	40–75	71.7	43.9	52.9	54.4
Lymphocytes	20–45	25.4	49.3	40.2	37.0
Monocyte	2–10	2.7	5.6	2.0	5.7
Eosinophils	1–6	0	0.6	0.2	2.7
Platelet count (per μL)	150,000–450,000	170,000	177,000	223,000	230,000
Red Cell Indices					
Mean Corpuscular Volume (MCV) (fl)	76.0–96.0	60.7	61.6	60.7	60.3
Mean Corpuscular Hemoglobin (MCH) (pg)	27.0–32.0	18.4	18.3	18.4	18.3
Mean Corpuscular Hemoglobin Concentration (MCHC) (%)	31.0–36.0	30.3	29.9	30.3	30.3
Red Blood Cell Distribution Width - Standard Deviation (RDW-SD) (fl)	36.0–46.0	34.3	34.3	33.8	33.7
Red Blood Cell Distribution Width -Variation Coefficient (RDW-CV) (%)	11.5–14.5	18.6	18.3	18.4	18.4
Sodium (mmol/L)	135–145	137.13			136.43
Potassium (mmol/L)	3.5–5.3	4.94			3.98
Chloride (mmol/L)	97–106	103.6			103.52
Anion Gap (mmol/L)	7.0–21.0	26.59			16.55
Creatinine (μmol/L)	53–106	11.88			68.59
C-reactive protein (CRP) (mg/dL)	0.01–0.50	10.12			0.71
D-Dimer (ng/mL)	<550	450.0			405.0
Fibrinogen (mg/dL)	180–350				381.7
Prothrombin Time					
Control (sec)					12.0
Patient (sec)	12–15				12.3
Index (%)					97.6
Ratio					1.0
INR					1.0
Anti-dengue IgG & IgM (ELISA)					
Dengue IgG		Negative for IgG			
Dengue IgM		Positive for IgM			
Blood for Culture and Sensitivity		No organism isolated in aerobic and microaerophilic condition at 35-degree Celsius over 72 h

Serum electrolytes and creatinine tested on admission revealed normal value, however, one inflammation marker, C-reactive protein (CRP) was 10.12 mg/dL. Blood for culture and sensitivity (CS) revealed no organism in aerobic and microaerophilic conditions at 35 °C in 72 h. Markers of coagulation were within normal limits for our case including D-Dimer, Fibrinogen, and Prothrombin time (Table 1).

*Clinical Diagnosis:* Based on the patient's history, clinical presentation and examination, and laboratory investigations, we diagnosed the patient as a case of COVID-19 Pneumonia with Dengue fever with Heterozygous Beta-thalassemia or beta-thalassemia trait.

*Treatment:* The patient was treated at prone positioning for at least 14–16 h a day from the beginning. Taking into account that, hematocrit in the patients aligned with the lower border and infected with dengue virus, mostly liquid diet was started along with intravenous Ringer's lactate (RL) solution at the rate of 80 ml/h (total 1920 ml) over the first 24 h of hospital admission before getting the laboratory investigation reports (Figure 4). Based on the initial CBC report with low MCV, MCH, MCHC, and RDW-SD, we further investigated to rule out hemoglobinopathies with Capillary Zone Electrophoresis and the result was revealed Beta Thalassemia (Heterozygote) or beta-thalassemia trait. Intravenous (IV) infusion remained at the same rate (80 ml/h) on day 2 with the addition of 500 ml of Glucose based Oral Rehydration Solution (G-ORS) (Figure 4). CBC was scheduled every 24 h to evaluate the response of hematocrit on fluid management and to check the number of platelets. Injection Ceftriaxone 2 g once daily and Ciprofloxacin 400 mg twice daily was started according to the protocol of the Dhaka Hospital for the management of COVID-19 patients for the treatment of possible bacterial coinfection after noticing high CRP on day 2. Low Molecular Weight Heparin (Enoxaparin 60 mg, subcutaneous injection twice daily) started to prevent possible coagulopathy from COVID-19 as per protocol [7]. Injection Omeprazole 40 mg IV once daily to manage stress ulcer and Domperidone 10 mg tablet 3 times daily before a meal, peroral added in medication list to minimize the event of a gastrointestinal upset of patient as the patient has a history of vomiting. Tablet Zinc 20 mg, Vitamin C 250 mg, Vitamin D 1000 IU, Famotidine 20 mg added as these have anti-viral properties. On day 3, RL infusion readjusted with 100 ml/h IV for the next 24 h with 1500 ml G-ORS plus an adequate liquid diet (Figure 4). To maintain the hematocrit level within the normal range, the intravenous infusion was continued to administer at 80 ml/h and 40 ml/h on day 4 and 5, respectively (Figure 4). On day 5, injectable antibiotics stopped as there was no growth isolated in blood CS. The patient got subcutaneous Enoxaparin for 7 days, then switched to oral Rivaroxaban 10 mg, once daily for the next 30 days.

## 4 Results

**Figure 3:**
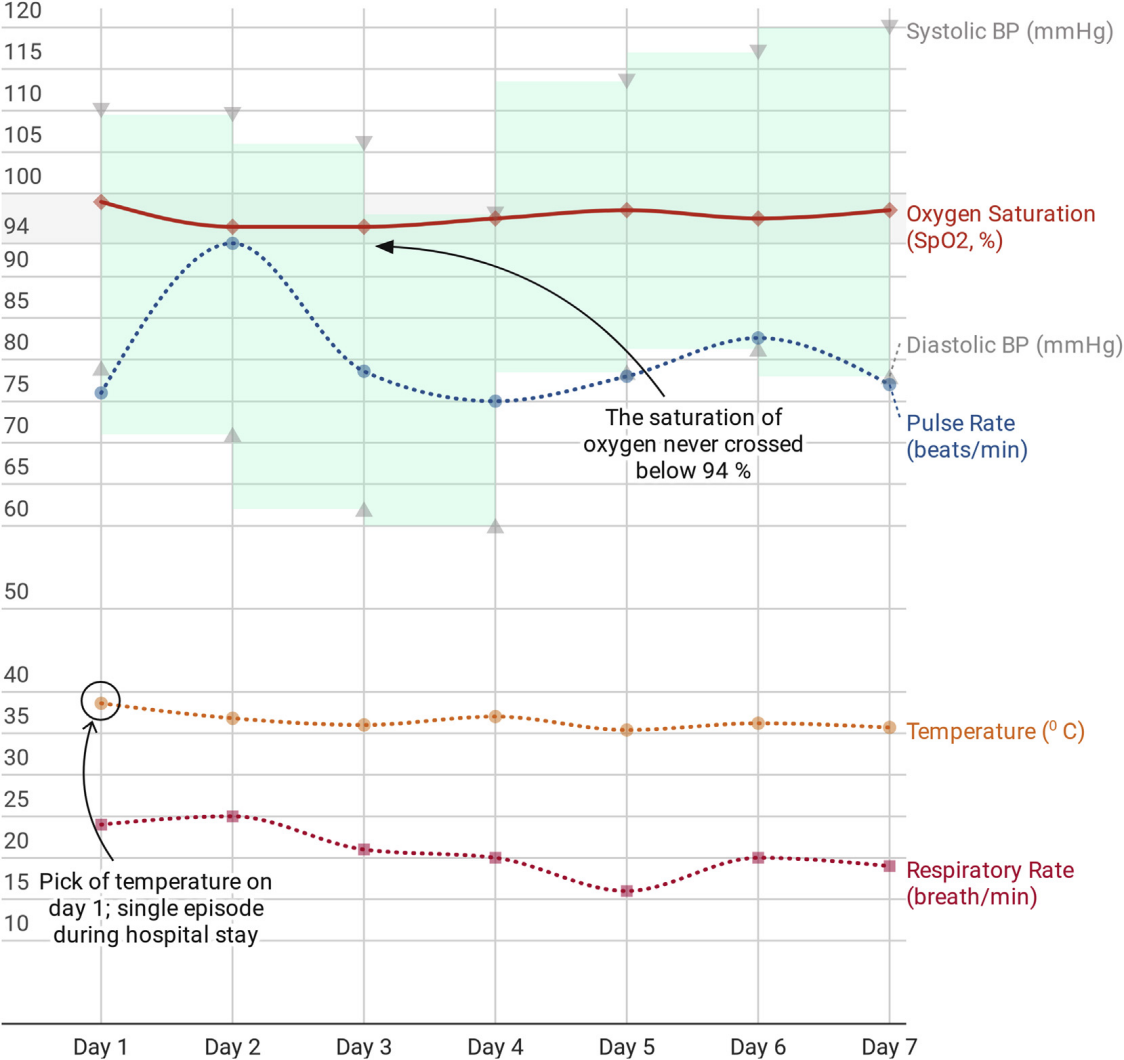
Vital signs during hospital stay [Interactive weblink: https://www.datawrapper.de/_/JtoUG/].

**Figure 4:**
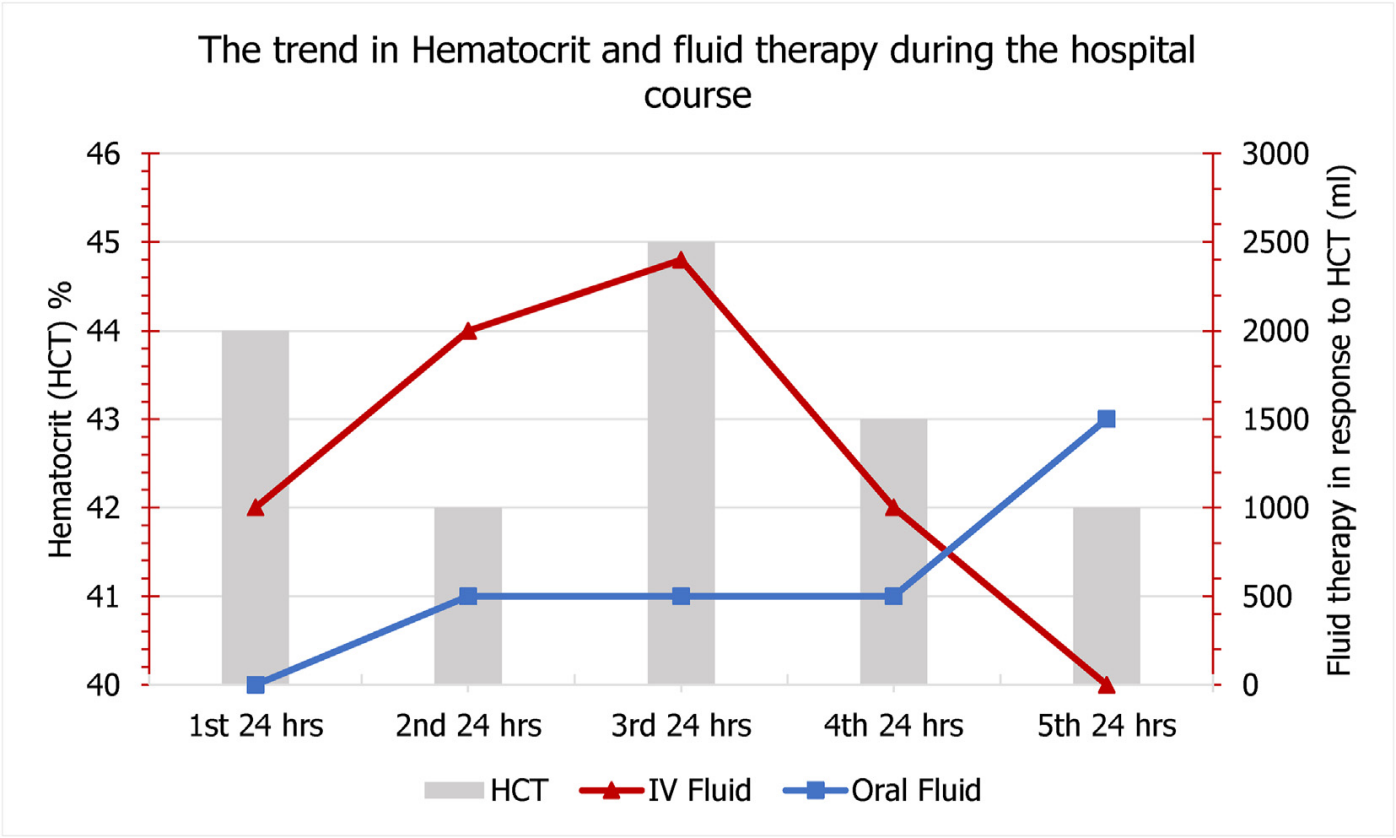
Daily Hematocrit (HCT) level (%) and fluid therapy (IV and oral, in ml) in response.

*Outcome and follow-up:* The patient had another episode of vomiting out of blood within the first 24 h of admission and also developed fever (single episode, without chills and rigor, temperature 39 °C) during that time and was given taped-sponging and then antipyretics (oral Paracetamol 500 mg 2 tablets once, at a time) accordingly. Fever subsided from the second day of admission. His oxygen saturation was well-maintained throughout his hospital stay, the respiratory pattern was regular and uneventful (Figure 3). The patient got a calculated amount of IV fluid (every 24 h, according to hematocrit levels) for the management of dengue (Figure 4). His BP, pulse rate and volume, intake-output chart, respiratory rate, temperature were monitored routinely (every 6 h apart, daily) (Figure 3). Throughout the period of the patient's hospital stay, the patient did not require oxygen support or steroids to prevent cytokines storm. During these events, the patient had just been diagnosed with the beta-thalassemia trait and was unaware of these genetic abnormalities. After 6 days of uneventful hospital stay, the patient was discharged on day 7 with the prescription including oral Rivaroxaban 10 mg, Famotidine 20 mg, Zinc 20 mg, Vitamin C 250 mg, and Vitamin D 1000 IU. The patient was advised to continue ongoing treatment at home isolation. At discharge, the patient was afebrile, euvolemic without any respiratory difficulty. During discharge, genetic counseling has been conducted, and advice for carrier screening of family members of hemoglobinopathies as well. During our follow-up, the patient became negative for SARS-CoV-2 on day 21. No further episodes of fever and vomiting after discharge from the hospital.

## 5 Discussion

Coinfection of SARS-CoV-2 with other respiratory viruses was previously reported on hospital patients from New York City and California, with incidences of 2.1% and 20.7% respectively [12, 13]. Notable two cases of COVID-19 and Dengue fever coinfection from Dengue epidemic regions like Thailand and Singapore have been identified to date from a comprehensive literature review and in our knowledge [14, 15]. Dengue fever has historically been most widespread in Southeast Asia and the Pacific Islands. Climate change and urbanization have a significant impact on the life cycle of Aedes mosquitos, which has an impact on the transmission of the dengue virus [16]. Similar to Thailand and Singapore, Bangladesh, a monsoon country, is also part of this region of South East Asia that is endemic to Dengue fever during this period (June through September) of the year [17].

Both Dengue and COVID-19 are viral infections and possess several similar features. So, a high level of suspicion on the background of dengue epidemiology and locality is important. Recent papers underlined the difficulties in distinguishing the non-severe case of Dengue from COVID-19 in dengue epidemic regions [18]. Another recent piece of evidence highlighted the need for the adoption of a rapid strategy to prevent a possible outbreak of dengue during the COVID-19 pandemic in Bangladesh [11]. Here we report a case of heterozygous beta-thalassemia (previously undiagnosed) or beta-thalassemia trait, coinfected with COVID-19 and dengue fever simultaneously and well managed at the COVID-19 unit of icddr,b.

Patients coinfected with SARS-CoV-2 and other respiratory viruses had clinical characteristics very similar to those of COVID-19 [19]. Conversely, mosquito-borne dengue fever is characterized by mild to moderate grade fever accompanied by vomiting, rashes, aches, and body-ache with the alarm sign as blood vomit [20, 21].Despite this, published case reports from different countries have found patients with similar clinical presentations and comparable laboratory findings in blood pictures, except for a frequently lowered platelet count in COVID-19 and Dengue coinfection [22, 23, 24, 25, 26], however in our case we found the platelet count was within normal range. There are concerns regarding potential cross-reactivity between these two viruses, serodiagnostic tests for Dengue virus may yield many false-positive results for non-severe SARS-CoV-2 and vice versa in dengue-endemic regions [27]. However, icddr,b serodiagnostic laboratory uses the Panbio™ Dengue Duo IgM Capture and IgG Capture ELISA which has a serological sensitivity of 94.7% and specificity of 100% [28].

Depending on the severity of coronavirus disease, our reported patient was a mild COVID-19 case, according to his chest x-ray score and symptoms presented [7, 9, 10]. Even though epidemiologic evidence concerning SARS-CoV-2 infection in patients with thalassemia and sickle cell disease is currently lacking, the COVID-19 pandemic represents a significant challenge for hemoglobinopathy patients, their families, and their attending physicians [29]. This is particularly true for patients living in developing or low-income countries, where disease-specific management programs are lacking and access to modern therapy is limited. The clinical impact of COVID-19 in hemoglobinopathy patients is not yet defined. In general thalassemia traits, and particularly Hemoglobin E (HbE), is protective against COVID-19 infection in similar ways to the numerous thalassemia traits conferring protection against malaria and the dengue virus [30]. Recent studies in thalassemia have pointed to the involvement of microRNAs (miRNAs) in malarial pathogenesis and anti-plasmodial defense. miRNAs can downregulate gene expression in translational repression and exhibit decisive regulatory functions associated with a variety of disease processes, including microbial defense. In dengue virus infection, which is perennially endemic in South-East Asia, red blood cell precursors in Thai carriers of thalassemia and HbE trait were significantly less susceptible to the dengue virus compared to normal controls [31]. The hypothesized conferred protection against COVID-19 could also derive from local SE Asian HLA-class allotypes, possibly in linkage disequilibrium with the thalassemia mutations. Protection from and resistance to severe malaria has been described in association with HLA antigens in the African continent [30]. However, no HLA associations with COVID-19 infection have been noted in a recent report. Greece and Cyprus with a high homogenous thalassemia carrier rate, seem to be experiencing lower COVID-19 rates compared to their European peers, even during the second wave. In Cambodia and Laos, the thalassemia prevalence is 62.7% (HbE being the most prevalent, at 56%) and also in Myanmar (HbE carrier rates vary between 1.9 and 42% depending on ethnic group and geography), low infection and death rates are also seen [32]. This viewpoint complies well with our recorded case that showed minimally any alarming COVID-19 related symptoms, such as hypoxemia and coagulopathy, although there is still controversy.

Sahu and Kumar underlined that populations with benign hematological disorders such as thalassemia (and its trait) should be given extra attention to avoid severe consequences and that family physicians and community health centers should be included in this effort [33]. The issues experienced by family physicians in Singapore during a twin outbreak of the COVID-19 pandemic and dengue-endemic were recently explored by Lam et al. [34] It is therefore recommended that community clinics screen and diagnose those coinfection cases with benign hematological disorders and refer them to a higher center to avoid a critical situation. Nevertheless, the possibility of coinfection of Dengue fever with a COVID-19 case may be considered with higher propriety in clinical management if any alarming sign such as unremitting fever supportive of dengue fever is evident especially during the Dengue fever epidemic. Essentially, fluid therapy requires to have a cautious approach to prevent probable adverse events in managing cases with such coinfections.

## Declarations

### Author contribution statement

All authors listed have significantly contributed to the investigation, development and writing of this article.

### Funding statement

This research did not receive any specific grant from funding agencies in the public, commercial, or not-for-profit sectors.

### Data availability statement

Data associated with this study has been deposited at Mendeley Data under the accession number doi:10.17632/65g5z7fpdc.4.

### Declaration of interests statement

The authors declare no conflict of interest.

### Additional information

This case report's preprint is available at SSRN through this link (https://dx.doi.org/10.2139/ssrn.3785263). No other additional information is available for this paper.
